# First Description of Natural and Experimental Conjugation between Mycobacteria Mediated by a Linear Plasmid

**DOI:** 10.1371/journal.pone.0029884

**Published:** 2012-01-03

**Authors:** Michelle Christiane da Silva Rabello, Cristianne Kayoko Matsumoto, Luiz Gonzaga Paula de Almeida, Maria Carmen Menendez, Rosangela Siqueira de Oliveira, Rosa Maria Silva, Maria Jesus Garcia, Sylvia Cardoso Leão

**Affiliations:** 1 Departamento de Microbiologia, Imunologia e Parasitologia, Escola Paulista de Medicina, Universidade Federal de São Paulo, São Paulo, Brazil; 2 Laboratório Nacional de Computação Científica, Petrópolis, Brazil; 3 Departamento de Medicina Preventiva, Facultad de Medicina, Universidad Autónoma de Madrid, Madrid, Spain; 4 Núcleo de Tuberculose e Micobacterioses, Instituto Adolfo Lutz, São Paulo, Brazil; University of Wisconsin, Food Research Institute, United States of America

## Abstract

**Background:**

In a previous study, we detected the presence of a *Mycobacterium avium* species-specific insertion sequence, IS*1245*, in *Mycobacterium kansasii.* Both species were isolated from a mixed *M. avium*-*M. kansasii* bone marrow culture from an HIV-positive patient. The transfer mechanism of this insertion sequence to *M. kansasii* was investigated here.

**Methodology/Principal Findings:**

A linear plasmid (pMA100) was identified in all colonies isolated from the *M. avium*-*M. kansasii* mixed culture carrying the IS*1245* element. The linearity of pMA100 was confirmed. Other analyses suggested that pMA100 contained a covalently bound protein in the terminal regions, a characteristic of invertron linear replicons. Partial sequencing of pMA100 showed that it bears one intact copy of IS*1245* inserted in a region rich in transposase-related sequences. These types of sequences have been described in other linear mycobacterial plasmids. Mating experiments were performed to confirm that pMA100 could be transferred *in vitro* from *M. avium* to *M. kansasii*. pMA100 was transferred by *in vitro* conjugation not only to the *M. kansasii* strain from the mixed culture, but also to two other unrelated *M. kansasii* clinical isolates, as well as to *Mycobacterium bovis* BCG Moreau.

**Conclusions/Significance:**

Horizontal gene transfer (HGT) is one of most important mechanisms leading to the evolution and diversity of bacteria. This work provides evidence for the first time on the natural occurrence of HGT between different species of mycobacteria. Gene transfer, mediated by a novel conjugative plasmid, was detected and experimentally reproduced.

## Introduction

Insertion sequences (ISs) are mobile genetic elements, capable of transposing and inserting at multiple sites in target DNA molecules [1]. The genus *Mycobacterium* contains a large number of different insertion elements, several of them found in members of the *Mycobacterium avium* complex (MAC) [2]. MAC organisms are ubiquitous in nature, and besides the classical species *M. avium* and *Mycobacterium intracellulare*, two new species were recently described: *Mycobacterium chimaera* [3] and *Mycobacterium colombiense* [4]. *M. avium* is responsible for opportunistic infections in animals and humans, and has been frequently associated with disseminated infections in HIV-positive patients [5]. The insertion sequence IS*1245* is a mobile element highly prevalent in subspecies of *M. avium: M. avium* subsp. *avium*, *M. avium* subsp. *hominissuis,* and *M. avium* subsp. *silvaticum*. Multiple IS*1245* copies are usually present in *M. avium* clinical isolates, making this repetitive sequence a useful tool for epidemiological studies [6], [7], [8], [9], [10]. However, some studies have shown that this insertion element is sporadically present in other mycobacterial species, suggesting that it may be dispersed by horizontal gene transfer (HGT) [11].

Recently, our group confirmed the presence of the IS*1245* element in colonies of *Mycobacterium kansasii* isolated from a *M. avium*-*M. kansasii* mixed culture from a bone marrow specimen of an HIV-positive patient [12]. Fifteen colonies were isolated from the original culture, four of which were identified as *M. avium* and 11 as *M. kansasii*. Eight of the 11 *M. kansasii* colonies generated IS*1245* amplicons by PCR. The presence of the IS*1245* element in these *M. kansasii* colonies was confirmed by IS*1245* amplicon sequencing and by detection of a 4,750-bp hybridization band in restriction fragment length polymorphism analysis using IS*1245* as the probe (RFLP-IS*1245*) [12]. The results obtained strongly suggested that *M. kansasii* acquired the IS*1245* element from the *M. avium* strain following an HGT event.

Conjugation, transduction and transformation are forms of HGT that occur naturally between bacteria. These three mechanisms have already been described or suggested in studies with mycobacteria, but information and experimental evidence are scarce. The majority of studies suggesting HGT in mycobacteria are based on comparative genome analyses [13]. Phages and plasmids were demonstrated to be the main vehicles of HGT and could contribute to IS dispersion among genomes. Many plasmids and bacteriophages have been described in the genus *Mycobacterium* [14], [15], [16] and some mycobacterial plasmids were previously shown to carry IS elements [15], [16]. Plasmid pCLP from *Mycobacterium celatum* contains sequences similar to resolvases and transposases found in the *Mycobacterium tuberculosis* genome [15]. Plasmid pMUM001 from *Mycobacterium ulcerans* carries 26 copies of various ISs and IS fragments, 12 of which are known elements present in the chromosome of *M. ulcerans* (4 copies of IS*2404* and 8 copies of IS*2606*) [16]. Thus, IS jumping between bacterial chromosome and plasmids does not appear to be a rare event.

In this study, we investigated the mechanism of IS*1245* element transfer from *M. avium* to *M. kansasii*, resulting in the detection of a 100-kb linear conjugative plasmid (pMA100).

## Results

### Identification and characterization of a novel linear plasmid (pMA100)

**Figure 1:**
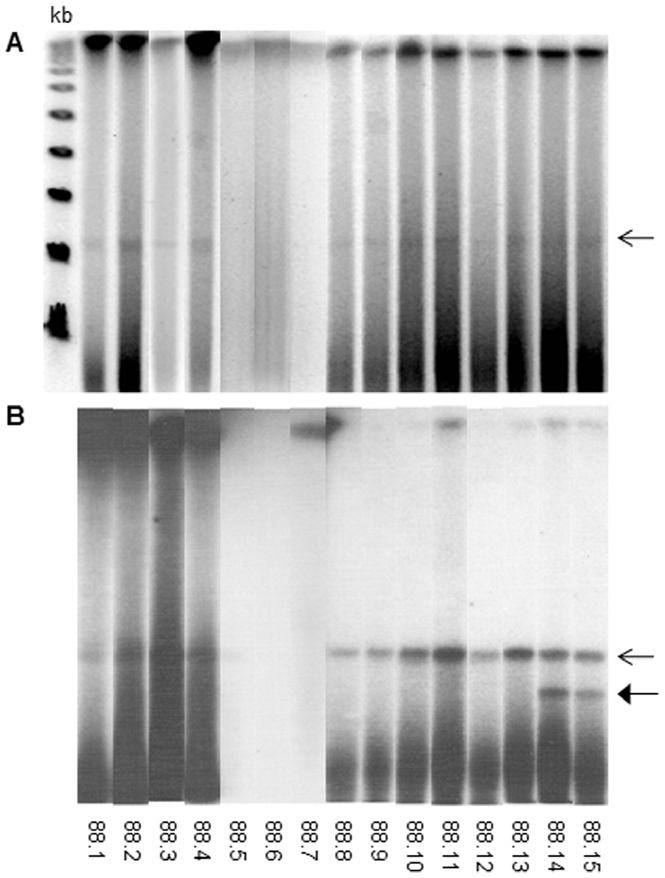
PFGE and Southern blot hybridization with IS*1245*-derived probe of ***M. avium*** **and** ***M. kansasii*** **colonies.** (A) PFGE with undigested DNA; (B) Southern blot hybridization with IS*1245*-derived probe. Open arrow indicates pMA100; closed arrow indicates the uncharacterized smaller hybridization band. 88.1 to 88.4 = *M*. *avium*; 88.5 to 88.75 = PCR−IS*1245-*negative *M*. *kansasii*; 88.8 to 88.15 = PCR−IS*1245-*positive *M. kansasii.* On the left, Lambda Ladder PFG Marker (NewEngland BioLabs) molecular size markers.

An extrachromosomal band of approximately 100 kb, designated pMA100, was identified by pulsed-field gel electrophoresis (PFGE) of undigested DNA with four *M. avium* colonies and eight of the 11 *M. kansasii* colonies from the original *M. avium-M. kansasii* mixed culture, all of which produced amplicons by PCR-IS*1245* (Figure 1A). The presence of IS*1245* in pMA100 was confirmed by Southern blot hybridization of pulsed-field gels using a radioactivity-labeled IS*1245* complementary probe (Figure 1B). Neither the 100 kb band nor any hybridization signal with the IS*1245*-derived probe was detected in the three *M. kansasii* colonies that did not generate IS*1245* amplicons (Figure 1A and 1B). A second hybridization band, undetected in PFGE gels stained with ethidium bromide, was observed with two of the eight PCR-IS*1245* positive *M. kansasii* colonies (88.14 and 88.15). This experiment was repeated and the results were reproduced (Figure 1B).

**Figure 2:**
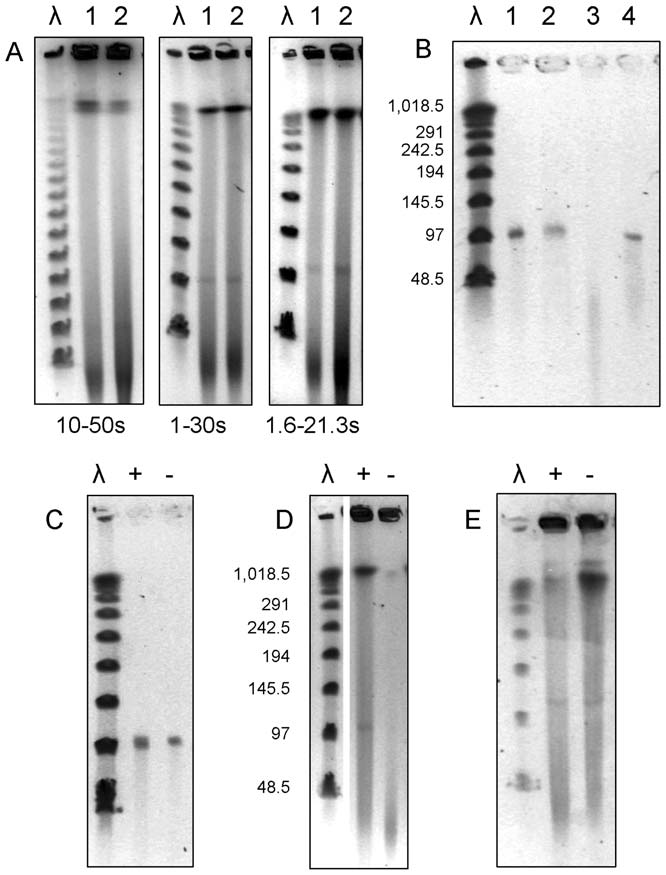
PFGE of DNA genomic preparations. (A) PFGE with undigested DNAs from *M. avium* 88.3 (1) and *M. kansasii* 88.8 (2) under different switch times, indicated below each figure; (B) pMA100 extracted from PFGE gels and treated with exonuclease III (3) or exonuclease lambda (4); (C) pMA100 extracted from PFGE gels and treated (+) or not (-) with topoisomerase I; (D) DNA prepared with (+) or without (-) adding proteinase K to the lysis buffer; (E) same as in (D) in PFGE gels and running buffer prepared with 0.2% SDS. λ: DNA concatemers of the bacteriophage λ genome.

pMA100 co-migrated with linear lambda concatemers in PFGE using three different switch times, suggesting that it was a linear molecule (Figure 2A). Experiments with exonuclease III, exonuclease lambda and topoisomerase I confirmed the linearity of this molecule. pMA100 was sensitive to exonuclease III (which degrades DNA from free 3′ ends), and its migration was not affected by topoisomerase I, an enzyme that relaxes circular plasmids. Moreover, it was resistant to the action of exonuclease lambda, which degrades DNA from free 5′ ends, suggesting that pMA100 may have covalently bound proteins at its terminals (Figure 2B and 2C).

In plugs prepared without the addition of proteinase K to the lysis buffer, pMA100 did not enter the gel preparation during PFGE, suggesting again the presence of covalently bound proteins at its terminals (Figure 2D). Moreover, pMA100 was able to penetrate the gel after addition of 0.2% SDS, which denatures terminal end proteins (Figure 2E).

### Partial sequencing of pMA100

**Figure 3:**
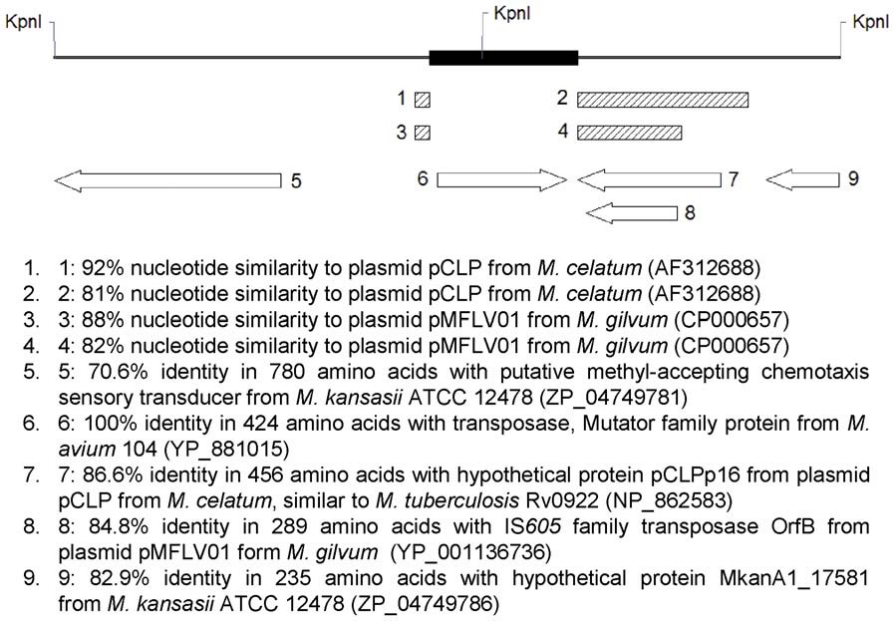
Gene features of the IS*1245* flanking regions in pMA100. The similarities identified in the 7,525-bp sequenced fragment of pMA100 are indicated. Similarities at nucleotide levels were identified using BLASTn and the regions of similarity are depicted as hatched bars; identities at amino acid level were identified using BLASTx and each region is shown as a white arrow, indicating the direction of transcription. Black bar localizes the IS*1245* element. KpnI restriction sites are indicated.

DNA from *M. kansasii* (colony 88.8) digested with KpnI and analyzed by RFLP-IS*1245* generated two hybridization fragments of approximately 3,500 bp and 4,100 bp, respectively (data not shown). Both fragments were cloned and sequenced. The analysis of the sequences obtained confirmed the presence of an intact copy of the IS*1245* element in pMA100. IS*1245* has a KpnI restriction site at position 499, and as a consequence, the first 499 bp of IS*1245* were located in the ∼4,100 bp KpnI fragment and 914 bp in the ∼3,500 bp KpnI fragment. The IS*1245* flanking region had >80% similarity to transposase-related genes present in other mycobacterial linear plasmids, namely the IS*605 orfB* related sequence present in plasmid pMFLV01 from *Mycobacterium gilvum* PYR-GCK (GenBank accession numbers NC_009339 and CP000657) and sequences related to Rv0922 and Rv0921 (IS*1535*) from *M. tuberculosis* present in plasmid pCLP from *M. celatum* [15]. The adjacent DNA sequences did not show similarity at the nucleotide level with other sequences in the GenBank database; however, it was possible to detect similarities at the amino acid level with two putative proteins described in the *M. kansasii* genome (Figure 3).

### pMA100 was transmissible by conjugation to *M. kansasii* and *Mycobacterium bovis* BCG

Different mating experiments were carried out using *M. avium* colony 88.3, bearing pMA100, as donor and different recipient strains: *M. kansasii* colony 88.6, *M. kansasii* IAL 413 *M. kansasii* IEC 6805, *M. bovis* BCG Moreau or *Mycobacterium smegmatis* mc^2^155. Table 1 shows the results of PCR-IS*1245* screening of 1,770 recipient strains colonies isolated after the mating experiments. Eight *M. kansasii* and 11 *M. bovis* BCG transconjugant colonies were detected by PCR-IS*1245*. No transconjugants were obtained when *M. smegmatis* mc^2^155 was used as recipient strain.

**Table 1:** Conjugation experiments using *M. avium* 88.3 as the donor strain.

recipient strain	conjugation conditions		screened recipient colonies	PCR-IS*1245* positive colonies
	incubation temp (°C)	time (days)		
*M. kansasii* 88.6	30	10	126§	1
	37	10	180§	3
*M. kansasii* IAL 413	37	10	75§	1
*M. kansasii* IEC 6805	37	10	150§	2
*M. bovis* BCG Moreau	37	*	39£	11
*M. smegmatis* mc^2^155	37	**	1200¶	0

*three experiments with 15, 20 and 25 days incubations, respectively.

**three experiments with 2, 4 and 10 days incubations, respectively.

§pigmented colonies after exposure to light.

£PCR-IS*6110* positive.

¶grown on LB agar.

**Figure 4:**
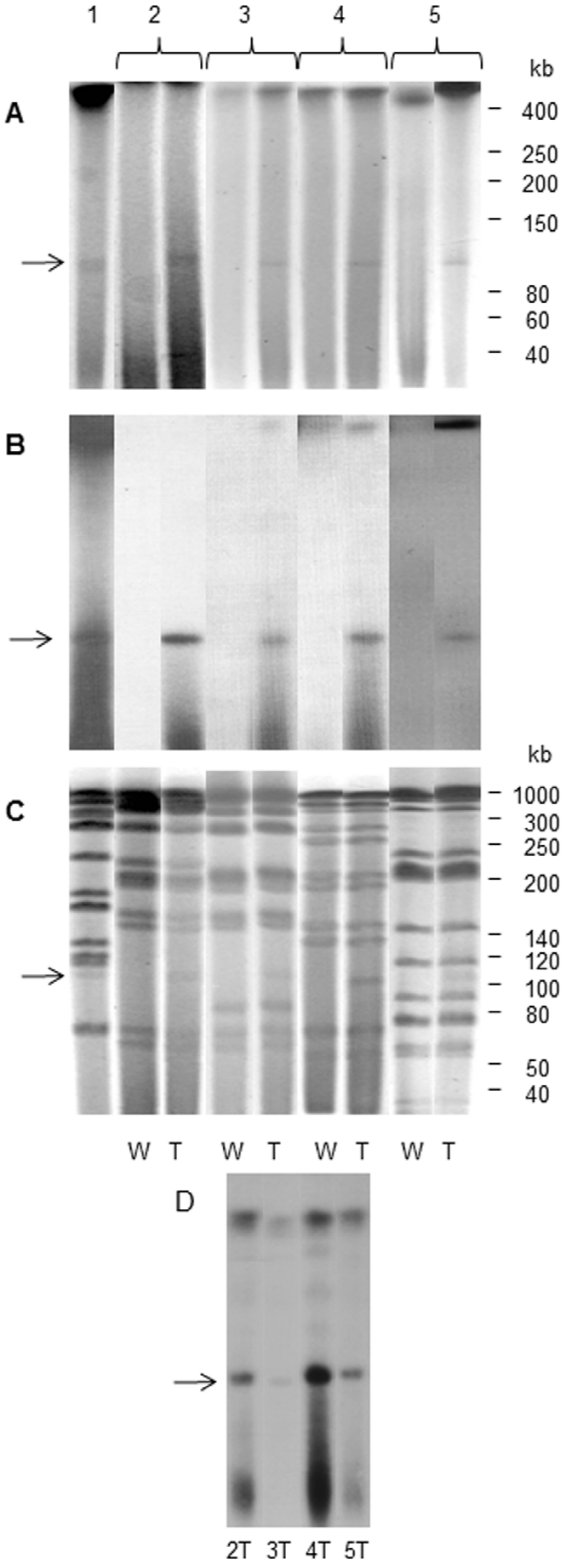
Analysis of transconjugants isolated in mating experiments using *M. avium* 88.3 as donor strain. (A) PFGE with undigested DNA; (B) Southern blot of PFGE gels with undigested DNA and hybridization with IS*1245*-derived probe; (C) PFGE-DraI; (D) Southern blot of PFGE-DraI gels and hybridization with pMA100-derived probe. Open arrows indicate the linear plasmid pMA100. 1: *M. avium* 88.3; 2: *M. kansasii* 88.6; 3: *M. kansasii* IAL 413; 4: *M. kansasii* IEC 6805; 5: *M. bovis* BCG Moreau; W: wild-type colony; T: transconjugant colony.

Additional experiments were carried out to confirm the presence of IS*1245* and pMA100 in the transconjugant colonies. In each case, the presence of the 100 kb plasmid band, which hybridized with IS*1245* was confirmed by PFGE (Figure 4A and 4B). Moreover, PFGE-DraI typing corroborated that each corresponding pair of recipient and transconjugant colonies belonged to the same strain (Figure 4C). Hybridization of DraI digested DNA from transconjugant colonies with the pMA100 derived probe was used to demonstrate that, besides the IS*1245* element, the pMA100 molecule was also transferred to *M. kansasii* and *M. bovis* BCG in the mating experiments and that pMA100 did not integrate into the recipient strains chromosome (Figure 4D).

No transconjugants were detected in mating experiments performed using the rapidly-growing mycobacterium *M. smegmatis* mc^2^155 as recipient. A total of 1,200 colonies from three mating experiments, identified as *M. smegmatis* for the ability to grow on LB agar in less than 7 days, were screened by PCR-IS*1425* but none produced IS*1245* amplicons (Table 1).

## Discussion

PFGE has been previously used for the detection of mycobacterial plasmids [17], [18], [19]. In the present study, PFGE allowed the identification of a 100-kb extrachromosomal band (pMA100) in PCR-IS*1245* positive *M. avium* and *M. kansasii* colonies from a mixed culture, suggesting that pMA100 could be a plasmid responsible for the natural transfer of the IS*1245* element between *M. avium* and *M. kansasii*.

This plasmid was shown to contain an intact copy of the IS*1245* element and was able to be transferred between *M. avium* and other slow-growing species, such as *M. kansasii* and *M. bovis* BCG. The weak intensity of bands corresponding to pMA100 in PFGE gels suggests that it may be a low-copy number plasmid.

A second smaller IS*1245*-hybridizing band was observed by Southern blot hybridization with two PCR-IS*1245* positive *M. kansasii* colonies (Figure 1B). It could be the same plasmid that underwent duplication and partial deletion. Otherwise, the IS*1425* element could have transposed to a second plasmid present in *M. kansasii*. This supplementary copy was not submitted to further investigation. The transposition of this IS during *in vitro* culture passages was previously described in *M. avium* [20], and in *M. kansasii* [12]. As PFGE requires recent cultures, it is possible that further transposition events occurred in some of these colonies when they were cultivated again. IS*1245* copies could have inserted in the chromosome.

The observation that pMA100 co-migrated with linear molecules in PFGE and the results obtained with exonucleases and topoisomerase I support the notion that the 100 kb molecule is linear and suggest an invertron-like structure. Besides, our results suggest that pMA100 could contain a protein covalently bound to the 5′ ends. This is a characteristic of linear elements with inverted repeats at their ends such as the linear plasmid pCLP from *M. celatum* [19], also found in some actinomycetes, viruses, bacteriophages [21]. These elements belong to a class of genetic elements called invertrons [21]. Conjugative linear plasmids have been identified in *Streptomyces* spp. [22], [23]. Because of this ability to conjugate, these elements can be spread among other actinomycetes, including mycobacteria and related organisms.

Partial sequencing of pMA100 confirmed that this plasmid contains an intact copy of IS*1245,* which is inserted in a transposase-related sequence homolog to *orf*B of IS*605* from *M. gilvum* plasmid pMFLV01 (acc. number: CP000657) and to a transposase identified in *M. celatum* plasmid pCLP (acc. number: AF312688). IS*605* is an insertion sequence reported frequently in strains of *Helicobacter pylori*, but has also been described in other bacteria [24]. The insertion of an IS within another IS is not a rare event, it has been described in *M. tuberculosis* [25] and other bacteria, such as *Pseudomonas syringae* [26], *Rhizobium meliloti* [27] and *Escherichia coli K-12* [28]. It has been previously shown that the genomic insertion loci of IS*1245* and IS*1311*, in *M. avium* strains carrying a single copy of any of these insertion sequences, are located in areas containing putatively truncated integrases and/or transposases [29]. Likewise, the transposase-related sequence identified in pMA100 could be a preferential locus for insertion of IS*1245*.

There are few studies and experimental evidence of HGT in the genus *Mycobacterium*. Reports on natural HGT in mycobacteria have been mostly based on circumstantial evidence, such as sequence similarities in phylogenetically distant organisms [15], [30], [31], [32]. Recent genetic and genomic studies have provided evidence of the contribution of HGT to the emergence of members of the *M. tuberculosis* complex and *Mycobacterium ulcerans* [33], [34], [35]. *M. ulcerans* is phylogenetically related to *Mycobacterium marinum* and is thought to have diverged from a common progenitor by acquisition of the virulence plasmid pMUM001 [35]. Genome sequencing of the rapidly-growing mycobacterium *Mycobacterium abscessus* CIP 104536^T^, revealed that this strain acquired a full-length prophage containing non-mycobacterial genes and also a mercury resistance circular plasmid (23 kb) almost identical to plasmid pMM23 from *M. marinum* [36], thus suggesting the occurrence of HGT among mycobacteria. Promiscuous mycobacterial plasmids with the ability to propagate among different members of MAC have been identified, and they could promote the acquisition of genetic material through HGT in nature [15], [37], [38].

Experimental studies have demonstrated HGT in mycobacteria in conjugation and transformation events artificially induced in the laboratory. Gormley and Davies obtained the conjugative transfer of a mobile plasmid from *Escherichia coli* to *M. smegmatis* [39]. Conjugation-mediated *Hfr* (high frequency recombination)-like transfer of chromosomal DNA was described in *M. smegmatis,* with a mechanism distinct from that observed in *E. coli Hfr* strains and not mediated by plasmids [40]. Bhatt *et al*. showed spontaneous transference of hybrids of *Streptomyces coelicolor* conjugative plasmid SCP2* and *Mycobacterium fortuitum* plasmid pAL5000 from *Streptomyces coelicolor* or *Streptomyces lividans* to *M*. *smegmatis* mc^2^155 in plate crosses. This transfer was DNase I sensitive and thus involved release of DNA from *Streptomyces* [41]. All these previous experiments involved only rapidly-growing mycobacterial species. The mating experiments described here demonstrated the transfer of the linear plasmid pMA100 and the IS*1245* element between slowly-growing mycobacteria.

We concluded that the most probable mechanism of acquisition of IS*1245* by *M. kansasii* and *M. bovis* BCG in mating experiments was conjugation. The possibility that this transference occurred by transformation or transduction was not completely ruled out yet but would be very unlikely.

Transformation would require competent bacteria. There are no reports of slowly-growing mycobacteria being naturally competent and we did not carry out any procedure to produce competent mycobacteria before the mating experiments. High-efficiency transformation has been a major limitation in the study of mycobacteria and the most successful method to transform mycobacteria is by electroporation, which was not used here. Spontaneous plasmid transformation was observed with rapidly-growing *M. smegmatis,* but was limited to small plasmids, which were taken up intact, while larger plasmids suffered deletions [41]. We showed here that pMA100 was not transferred to *M. smegmatis*. It seems highly improbable that a linear molecule of ∼100 k that might contain proteins attached to its ends could be easily and repeatedly transferred by natural transformation.

Transfection would require the presence of lytic phages and we never detected lytic plaques in any of our plates (data not shown). Repeated phage transduction of a unique 100 kb plasmid to different recipient bacteria would be highly unlikely. Otherwise, if the 100 kb molecule corresponded to phage DNA, it would have to integrate in the chromosome (lysogenic cycle) in order to be stably maintained in the recipient strains, and therefore would not be detected as an extrachromosomal molecule in PFGE gels, as shown in Figure 4.

Moreover, we have demonstrated that pMA100 is a linear molecule with an invertron-like structure. Invertrons were shown to be responsible for conjugation transfer in *Rhodococcus* [42], [43], [44] and *Streptomyces* [45], [46]. Similarly to *Mycobacterium*, both genera belong to the class Actinobacteria.

According to the number of transconjugants carrying IS*1245* in the mating experiment, the transfer frequency of pMA100 appears to be high under laboratory conditions. It is noteworthy that no transconjugants were obtained in mating experiments performed with *M. smegmatis* as the recipient bacterium. The analysis of more than 1,000 *M. smegmatis* colonies in mating experiments, suggests that this plasmid is not transferable from slow-growing to rapid-growing mycobacteria. Even though this conclusion needs further confirmation, it may indicate that conjugative mechanisms could differ between slow- and rapid-growing mycobacteria.

In conclusion, natural and experimental conjugation-mediated HGT between different species of slow-growing mycobacteria was demonstrated here for the first time. Details of the conjugation mechanism are still to be investigated. The presence of the IS*1245* element in the conjugative plasmid pMA100 is one of the possible molecular mechanisms to explain the distribution of this insertion sequence in mycobacterial species other than *M. avium*.

The complete sequencing of this linear plasmid is in progress. Sequence analysis could generate important information about genes involved in the mechanism of horizontal transfer in mycobacteria. Moreover, it has the potential to expand our understanding of how these acquired genes may influence the virulence, evolution and genetic diversity of mycobacteria. Finally, molecular tools could be developed from this conjugative linear plasmid, which could be used in genetic studies in mycobacteria, particularly with the avirulent strain *M. bovis* BCG.

## Materials and Methods

### Bacterial isolates and strains

Fifteen colonies, named 88.1 to 88.15, used in this study, were isolated from a *M. avium-M. kansasii* mixed culture obtained from a single bone marrow specimen from an HIV-positive patient, as described in a previous study [12]. Four colonies (88.1. to 88.4) were identified as *M. avium* and 11 as *M. kansasii* by phenotypic characteristics (growth rate and pigment production) and by PCR-restriction enzyme analysis of the 16S–23S internal transcribed sequence (PRA-ITS) [47] (Table 2). The 11 *M. kansasii* colonies showed indistinguishable PFGE-DraI patterns, except for the presence of a band of approximately 100 kb in eight of the 11 isolates and were, in fact, the same strain [12]. Two *M. kansasii* clinical isolates, IAL 413 and IEC 6805, *Mycobacterium smegmatis* mc^2^155, *Mycobacterium bovis* BCG Moreau and *Escherichia coli* DH5α (Life Technologies Co., Carlsbad, CA) were used in this study (Table 2).

**Table 2:** Clinical isolates and strains used in this study.

species	isolates/strains	PCR-IS*1245*
*Mycobacterium avium*	88.1 to 88.4	+
*Mycobacterium kansasii*	88.5 to 88.7	-
*Mycobacterium kansasii*	88.8. to 88.15	+
*Mycobacterium kansasii*	IAL 413	-
*Mycobacterium kansasii*	IEC 6805	-
*Mycobacterium smegmatis*	mc^2^155	-
*Mycobacterium bovis*	BCG Moreau	-
*Escherichia coli*	DH5α	-

(+) amplification of the 427-bp fragment; (-) no amplification.

Mycobacteria were cultivated in Middlebrook 7H9 liquid medium supplemented with albumin, dextrose and catalase (7H9-ADC) (Becton Dickinson, Franklin Lakes, NJ) or in Middlebrook 7H10 solid medium supplemented with oleic acid, albumin, dextrose and catalase (7H10-OADC) and stored at −80°C until use. Luria-Bertani (LB) liquid or solid medium [48] were used to cultivate *M. smegmatis* mc^2^155 and *E. coli*. Mycobacterial genomic DNA was extracted from a loop full of culture grown on 7H10-OADC as described by van Soolingen *et al.* [49].

### PCR-IS*1245*

A 427-bp fragment of the IS*1245* element (sequence positions: 197 to 623 - accession number: L33879) was amplified using primers P1 (GCCGCCGAAACGATCTAC) and P2 (AGGTGGCGTCGAGGAAGAC), as described by Guerrero *et al*. [6].

### PCR-IS*6110*

A 123-bp fragment of the IS*6110* sequence (sequence positions: 762 to 883 - accession number: X17348) was amplified using primers IS1 (CCTGCGACGTAGGCGTCGG) and IS2 (CTCGTCCAGCGCCGCTTCGG) as described by Eisenach *et al.* [50].

### PCR-pMA100

A 206-pb fragment of the sequenced region of pMA100 (sequence positions: 3029 to 3235 – accession number JN560941) was amplified with primers pMA100-1 (ACTCTCGGCTTCCTGTGTTG) and pMA100-2 (AGCGAAGTTCACCTTGGATG) with the same PCR conditions used to amplify a fragment of the IS*1245* element.

### Pulsed-field gel electrophoresis (PFGE)

High molecular weight DNA was prepared as described by Viana-Niero *et al*. [51] with some modifications. Bacteria were cultivated in 7H9-ADC at 37°C to an OD_650_ of 1–1.2. Cells were pelleted and frozen at −80°C for 1 h. After thawing, pellets were suspended in STE buffer (100 mM NaCl, 10 mM Tris-HCl, pH 8.0, 50 mM EDTA) with 0.1% Tween 80. The suspension was mixed with an equal volume of 1% low-melting preparative agarose (Bio-Rad Laboratories Inc., Hercules, CA) in 125 mM EDTA pre-warmed to 55°C, and the mixture was cast into plug molds. The plugs were treated overnight with 2 ml of 10 mg.ml^−1^ lysozyme in STE and incubated at 4°C for 1 h in 0.5 M EDTA plus 1% Sarkosyl. Proteinase K (RBC Bioscience Co., Taipei, Taiwan) was added at a final concentration of 2 mg.ml^−1^, and the plugs were incubated at 55°C for 24 h and then at 4°C for 1 h. Plugs were washed with 1X Tris-EDTA (TE: 10 mM Tris-HCl pH 8.0, 1 mM EDTA) and incubated in 1X TE containing 0.12 mg.ml^−1^ phenylmethylsulfonyl fluoride (Sigma-Aldrich Corp., St. Louis, MO) for 1 h at 55°C. Plugs were washed with 1X TE and stored in 0.5 M EDTA at 4°C until used. Plugs were washed in 0.05 M EDTA and loaded on 1% pulsed-field-certified agarose gels (Bio-Rad) in 0.5 X TBE buffer (45 mM Tris-HCl, 45 mM boric acid, 1 mM EDTA, pH 8.0). PFGE was carried out in a CHEF-DR III system (Bio-Rad) at 14°C for 24 h at 6 V.cm^−1^ with switch times of 1.6 to 21.3 s. Bacteriophage lambda ladder PFG marker (New England BioLabs, Ipswitch, MA) was used as molecular standard. For digestion, plugs were extensively washed in TE and incubated with DraI (Promega, Madison, WI) at 37°C overnight.

### Southern blot hybridization

After PFGE, DNA was blotted onto nylon membranes (Hybond-N-plus; GE Healthcare, Little Chalfont, UK) and probed with an IS*1245* complementary probe, prepared by PCR with primers P1/P2 with DNA from *M. avium* ATCC 25291^T^ and purified with QIAquick PCR Purification kit (Qiagen, Valencia, CA), and labeled with [α−32^P^]dCTP with Ready-To-Go DNA Labeling Beads (GE Healthcare), according to manufacturers' instructions.

### Characterization of pMA100

Experiments were carried out to determine if the pMA100 molecule was linear or circular. First, its migration in PFGE under three different switch times, 1.6 to 21.3 s, 1 to 30 s, and 10 to 50 s, was evaluated [52].

Besides that, the pMA100 band excised from PFGE agarose gels was treated in separate experiments with 30 U exonuclease III, 30 U exonuclease lambda or 30 U topoiosomerase I (all enzymes from New England BioLabs) for 3 h at 37°C, according to the manufacturer's protocols. The reactions were stopped by adding 50 mM EDTA and cooling to 4°C for 15 min, and the results were evaluated by PFGE.

In separate experiments, proteinase K was omitted during preparation of the plugs to investigate whether pMA100 contained a covalently bound protein. DNA treated or not with proteinase K was subjected to PFGE or to sodium dodecyl sulfate (SDS)-PFGE, i.e., after adding SDS (final concentration 0.2%) to both buffer and agarose gel preparation.

### Cloning and sequencing of KpnI restriction fragments containing the IS*1245* element

DNA from *M. kansasii* colony 88.8 was digested with KpnI at 37°C for 2 h, and subjected to RFLP-IS*1245,* performed as described by van Soolingen *et al*. [49]. The sizes of fragments hybridizing with the IS*1245* probe were calculated using the BioNumerics program version 5.1 (AppliedMaths, Sint-Martens-Latem, Belgium). Agarose gel slices containing the selected KpnI bands were excised from a second gel, and DNA purified using the Gel Extraction Kit (Qiagen), and ligated to pBluescript SK+ (Stratagene, Santa Clara, CA) digested with KpnI and dephosphorylated (CIAP; Life Technologies). Competent *E. coli* DH5α cells were transformed with the ligation products, and recombinant plasmids were selected on LB-agar plates containing 100 µg.ml^−1^ ampicillin, 0.25 mM IPTG and 1 mM X-Gal (Life Technologies). Colony lifts were carried out on Whatman 541 filters and were hybridized with the IS*1245*-derived probe, labeled with [α−^32^P]dCTP, according to the protocol described by Maas [53]. *E. coli* DH5α transformed with the plasmid pMA12, which contains the 427-bp fragment of IS*1245,* was used as the positive control of hybridization [49].

Recombinant plasmids were purified from positive colonies, identified after exposure to autoradiographic films, using Qiaprep Spin miniprep kit (Qiagen), and the inserts were sequenced using M13 primers and BigDye Terminator Cycle Sequencing Ready Reaction kit (Life Technologies) in an automated ABI Prism 377 sequencer (Life Technologies). Sequences were compared against the GenBank database using BLAST (http://www.ncbi.nlm.nih.gov/blast).

The obtained sequence was deposited under GenBank accession number JN560941.

### Mating experiments

Mating experiments were performed using *M. avium* 88.3 from the *M. avium-M. kansasii* mixed culture, bearing pMA100, as donor. Three *M. kansasii* strains, colony 88.6 (isolated from the mixed culture), IEC 6805 and IAL 413, *M. smegmatis* mc^2^155, and *M. bovis* BCG Moreau were used as recipients. All recipient strains were negative for the presence of both IS*1245* and pMA100, as assessed by PCR-IS*1245* and Southern blot hybridization of pulsed-field gels with the IS1245 derived probe, respectively.

Mating experiments were carried out on 7H10 agar plates as described by Parsons *et al*. [54] with some modifications. Donor and recipient strains cultures in 7H9-ADC liquid medium were incubated on a shaker at 37°C until DO600∼1.2 was reached. Aliquots of 0.5 ml of each culture were centrifuged at 14,000×g for 2 min and pellets were suspended in 0.5 ml of 7H9-ADC. Each mating pair suspension was combined and filtered through sterile 0.45 µm membranes (Millipore, Billerica, MA). These filters were incubated on 7H10-OADC plates at 37°C or 30°C. After different mating periods, the filters were transferred to sterile containers and washed with 7H9-ADC. Bacteria were resuspended in 1 ml of 7H9-ADC, and the suspensions were diluted and plated on 7H10-OADC (when *M. kansasii* or *M. bovis* BCG was used as the recipient strain) or LB (when *M. smegmatis* was the recipient strain) agar plates to obtain isolated colonies.

No selective medium was used to screen transconjugants, because no selection marker has been identified so far in pMA100, and a mixture of donor, recipient and transconjugant colonies was recovered on the 7H10-OADC plates. Only isolated colonies showing the characteristics of the recipient strain were used for screening of transconjugants by PCR-IS*1245*. *M. kansasii* colonies were identified by the production of yellow pigment after exposure to light. *M. bovis* BCG colonies were identified by PCR-IS*6110*. When *M. smegmatis* was used as recipient, the mating mixture was plated on LB agar, where *M. avium* cannot grow, and only *M. smegmatis* colonies were recovered.

Recipient colonies that generated IS*1245* amplicons after the mating experiments were assumed to be transconjugants.

The presence of plasmid pMA100 and IS*1245* in transconjugants was confirmed by PFGE and hybridization with the IS*1245*-derived probe and with a probe derived from the pMA100 sequenced region, labeled with [α−^32^P]dCTP. Each transconjugant was compared to the corresponding wild type strain by PFGE with DraI digested DNA.
